# Spectromicroscopy Study of Induced Defects in Ion-Bombarded Highly Aligned Carbon Nanotubes

**DOI:** 10.3390/nano14010077

**Published:** 2023-12-27

**Authors:** Sammar Tayyab, Alice Apponi, Maria Grazia Betti, Elena Blundo, Gianluca Cavoto, Riccardo Frisenda, Nuria Jiménez-Arévalo, Carlo Mariani, Francesco Pandolfi, Antonio Polimeni, Ilaria Rago, Alessandro Ruocco, Marco Sbroscia, Ravi Prakash Yadav

**Affiliations:** 1 Dipartimento di Fisica, Sapienza Università di Roma, Piazzale Aldo Moro 2, 00185 Rome, Italy; 2 Istituto Nazionale di Fisica Nucleare Sezione di Roma, Piazzale Aldo Moro 2, 00185 Rome, Italy; 3 Dipartimento di Scienze, Università Degli Studi Roma Tre and Istituto Nazionale di Fisica Nucleare Sezione di Roma Tre, Via della Vasca Navale 84, 00146 Rome, Italy

**Keywords:** carbon nanotubes, ion bombardment, SEM, XPS, Raman

## Abstract

Highly aligned multi-wall carbon nanotubes were investigated with scanning electron microscopy (SEM), Raman spectroscopy and X-ray photoelectron spectroscopy (XPS) before and after bombardment performed using noble gas ions of different masses (argon, neon and helium), in an ultra-high-vacuum (UHV) environment. Ion irradiation leads to change in morphology, deformation of the carbon (C) honeycomb lattice and different structural defects in multi-wall carbon nanotubes. One of the major effects is the production of bond distortions, as determined by micro-Raman and micro-X-ray photoelectron spectroscopy. We observe an increase in sp${}^{3}$ distorted bonds at higher binding energy with respect to the expected sp${}^{2}$ associated signal of the carbon 1s core level, and increase in dangling bonds. Furthermore, the surface damage as determined by the X-ray photoelectron spectroscopy carbon 1s core level is equivalent upon bombarding with ions of different masses, while the impact and density of defects in the lattice of the MWCNTs as determined by micro-Raman are dependent on the bombarding ion mass; heavier for helium ions, lighter for argon ions. These results on the controlled increase in sp${}^{3}$ distorted bonds, as created on the multi-wall carbon nanotubes, open new functionalization prospects to improve and increase atomic hydrogen uptake on ion-bombarded multi-wall carbon nanotubes.

## 1. Introduction

Since the pioneering work by Iijima on carbon nanotubes [1], multi-wall carbon nanotubes (MWCNTs) have generated enormous interest among scientists and engineers in the field of materials science [2]. These structures feature a set of unique properties due to small dimensions, closed topology and lattice helicity. The presence of atomic-scale defects in MWCNTs is responsible for changing or altering their mechanical and electronic properties [3]. Thus, the development of methods capable of controlling the amount and type of defects in MWCNTs is highly desirable, both for addressing the defect origin and for exploiting their properties to engineer the system characteristics. Among such methods, the use of heavy ions cause surface roughening and the removal of carbon atoms, leading to changes in the surface structure and properties of the nanotubes [4]. On the other hand, implanting lighter ions can affect the chemical composition and electronic properties of the nanotubes [5]. Both heavy and light ion bombardment can introduce vacancies and defects in the MWCNTs structure. These defects can act as active sites for chemical reactions or serve as traps for gas molecules, making them useful for various applications. We underline that functionalized MWCNTs can be applied in the fields of sensors [6,7] and biological sensors [8], composites [9], devices [10], energy storage systems [11,12], engineering [13], field effect transistors [14,15], power electronic devices [16], supercapacitor [17], etc.

Recent theoretical and experimental studies on the irradiation of carbon nanotubes with energetic particles have revealed a broad range of new interesting phenomena. Different ions like nitrogen [18], argon [19], helium [20], carbon and silver [21], with high [22] and low doses [23], and with varying energies, were used to bombard CNTs. These works aimed to induce defects, creating molecular junctions between the nanotubes [24], to form multiple species at the surface, to find a saturation damage, and to evaluate the stability and the evolution of the bonding. Effects on CNTs upon ion irradiation can be compared with analogous studies on its basic constituent, graphene. Carbon [25], helium [4], hydrogen [26] and deuterium were irradiated on graphene to functionalize it and to observe the charge carrier transport phenomena, to produce defects that lead to engineering process [27] and to unveil the band gap opening [26], useful for optoelectronic properties.

However, a quantification of the changes in hybridization and structure of MWCNTs that will eventually lead to a change in their electronic and physical properties, obtained by bombarding them with a sequence of non-interacting gases, remains an open question. To this purpose, we have investigated the influence of noble ion beams with different masses, at a fixed energy and given flux of ions. The choice of ion species, energy, and dosage, as well as the conditions under which the bombardment is conducted, can significantly influence the outcomes.

The main objective of the present study is to produce controlled defects on MWCNTs, in order to understand whether the different noble ion masses influence the quality and density of effects. Thus, we use heavy and light ions of noble gases (Ar${}^{+}$, Ne${}^{+}$ and He${}^{+}$) to check the suitability for applications requiring controlled modifications and functionalization of MWCNTs. In particular, we present a careful characterization of produced defects in ultra-high-vacuum (UHV) conditions, offering an ultra-clean environment. The achievement of a controlled density of defects mainly associated with sp${}^{3}$ hybridized levels in the C-atomic mesh of the CNTs would constitute active sites for CNTs functionalization. The increase in sp${}^{3}$ bonds is an important prerequisite for perspective usage, like improving alkali metal adsorption [28] for charge accumulation applications, or favoring hydrogen uptake [26] towards a potential solid-state material for hydrogen storage.

## 2. Materials and Methods

### 2.1. CNT Growth by Chemical Vapor Deposition

Vertically aligned MWCNTs were grown by the chemical vapor deposition (CVD) method with a home-made thermal CVD reactor in UHV, at the TITAN laboratory in Sapienza University of Rome, as described elsewhere [29,30]. Silicon was used as growth substrate, with a 2 μm thick buffer layer of SiO${}_{2}$. Electron beam evaporation was exploited over the substrate to deposit a 3 nm-thick layer of Fe catalyst. Samples were mounted inside a high vacuum reaction chamber with a base pressure in the low 10${}^{-7}$ mbar range and a two stage CNT synthesis was performed. First, we annealed the substrate for 4 min at 720 °C in a H${}_{2}$ atmosphere, necessary to remove the oxide components of catalyst that possibly stick to the surface of the Fe/SiO${}_{2}$/Si substrate. Annealing also helps to activate the catalyst layer (dewetting of catalyst) and the nucleation of iron-based nanoparticles. Next step leads to the growth of carbon nanotubes, in which the reaction temperature was increased to 740 °C and a carbon precursor (acetylene) was introduced inside the CVD chamber at a 300 sccm flow rate, without any carrier gas, up to a partial pressure of about 50 mbar. The growth time is limited to 10–12 min, in which reaction between carbon precursor and iron nanoparticles occurs and nanotubes grow vertically on the substrate by lifting the catalyst upward. After cooling, the vertically aligned (VA) MWCNTs grown on the Si substrate are extracted from the UHV chamber.

### 2.2. High Resolution Imaging

Scanning Electron Microscopy (SEM) measurements were carried out at CNIS Laboratory of Sapienza University Rome, with a field emission Zeiss Auriga 405 instrument, with a resolution of 1 nm at maximum magnification, by using a beam energy of 19 keV with a working distance in the 1.5–3.5 mm range.

### 2.3. X-ray Photoelectron Spectroscopy

The VA-MWCNTs samples were transferred to the integrated X-ray Photoelectron Spectroscopy (XPS)/micro-Raman apparatus at the SMART Laboratory [31] of the Department of Physics of Sapienza University, to carry out high-resolution XPS measurements, in UHV with a base pressure in the low 10${}^{-11}$ mbar range. Pristine MWCNTs samples (∼1 cm${}^{2}$ size) were mounted on the sample holders in such a way to be able to measure both the top and lateral faces of the nanotubes (see Figure S5 in the Supplementary Information, SI), and introduced into the preparation chamber.

For the XPS measurements, core-level photoelectrons were excited by a monochromatized X-ray Al K${}_{\alpha }$ (1486.8 eV) photon source (model SPECS XR50 MF) with focused beam, and analyzed by a PHOIBOS 150 analyzer (SPECS group, Berlin, Germany) working with 0.4 eV energy resolution, analyzed in constant pass energy (PE) mode set at 20 eV. Best spatial resolution of this setup was 30 μm. The electron binding energy (BE) scale was calibrated by using a gold foil that was in electrical contact with the sample, by acquiring the Au 4f${}_{7/2}$ core-level at 84.0 eV. The C 1s core level was taken in the (280–296) eV binding energy range.

### 2.4. Raman Spectroscopy

Micro-Raman (μ-Raman) measurements were performed at ambient conditions, with the sample being mounted on piezoelectric motors (by Attocube). The excitation laser was provided by a single frequency Nd:YVO${}_{4}$ laser (DPSS series by Lasos) emitting at 532.2 nm. The laser light was focused on the sample by a 50× long-working-distance objective with numerical aperture NA = 0.5 (by Olympus), resulting in a spot of about 1 μm. The same objective allowed us to collect the scattered light, in a backscattering configuration. The laser light was filtered out by a very sharp long-pass Razor edge filter (by Semrock). The Raman signal was spectrally dispersed by a 75 cm focal length Acton monochromator (by Princeton Instruments) equipped with a 300 grooves/mm grating, and was detected by a back-illuminated N${}_{2}$-cooled Si CCD camera (100BRX by Princeton Instruments). For depth-dependent Raman measurements, the laser was focused on the sample side. The laser spot was initially positioned at the very top of the carbon nanotubes (depth = 0) and the sample was then moved relative to the spot on-demand in order to have the laser focused at increasing depth along the nanotubes direction, going towards the sample substrate. Raman spectra were taken in the (500–3800) cm${}^{-1}$ wavenumber range.

### 2.5. Ion Bombardment on MWCNTs

Samples were measured at room temperature (RT) before annealing, to analyze the contamination content (O 1s core level signal at 6%). To remove the oxygen contamination [32] we annealed the pristine sample at 400 °C for 1 h reducing it to less than 1%. To avoid temperature healing effects, we avoided annealing the samples after ion bombardment. The ion bombardment was carried out with a sputtering ion source apparatus by Omicron Nanotechnology (ISE 10) on pristine MWCNTs samples coming from same batch of growth. Samples were bombarded with 3 keV Ar${}^{+}$, Ne${}^{+}$ and He${}^{+}$ ions impinging from a direction parallel (TOP side) and perpendicular (LAT side) to the CNT axis. We used an ion current density of 10 μA/cm${}^{2}$ and bombarded for 2400 s time, corresponding to a total number of 1.5 × 10${}^{17}$ bombarding ions.

## 3. Results and Discussion

### 3.1. SEM Analysis

**Figure 1:**
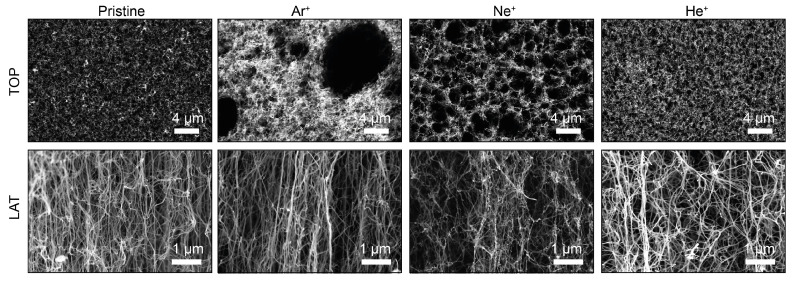
SEM image from the top side (TOP) and from the lateral side (LAT) of the CNTs; pristine (left), irradiated by 3 keV Ar${}^{+}$ (center-left), Ne${}^{+}$ (center-right) and He${}^{+}$ (right) ion beams.

The scanning electron microscopy images of the pristine MWCNTs displayed in Figure 1 (left), show the vertically aligned forests of MWCNTs grown on the Si substrate. At this high resolution spatial scale, a waviness in the orientation is visible that does not affect the average vertical alignment. On the top, a crust of twisted nanotubes of the order of few 10 nm is present, in agreement with previous observations on VA-CNTs [29]. All MWCNTs samples used in this experiment present heights of about 200 μm and diameters of about 10 nm, more details in the SI (Figure S1).

Ion bombardment with noble gas ions of different masses at the same energy (3 keV) affects both sides (top, [TOP] and lateral, [LAT]) of the samples. SEM images reveal that bombardment damages the surface of MWCNTs as shown in Figure 1, causing a rearrangement of the MWCNTs, with creation of holes across the entire surface due to coalescence in bundles, clusterization by combination of several CNTs and interlacing of external walls of individual CNTs with each other. From an analysis of the SEM images, we can extract the mean hole area by applying a grain analysis based on a watershed algorithm [33], implemented in the image-processing software Gwyddion (for more details see the SI Figure S2). Through this analysis we can find the area of the holes present in the TOP SEM image of each differently bombarded sample, that results to be roughly 0.07 ± 0.04 μm${}^{2}$ (pristine), 0.09 ± 0.04 μm${}^{2}$ (He${}^{+}$), 2.8 ± 1.4 μm${}^{2}$ (Ne${}^{+}$) and 5.5 ± 2.6 μm${}^{2}$ (Ar${}^{+}$) (see SI Figure S3). We observe that the larger the ion mass, the larger the mean hole size induced on the sample. However, the He${}^{+}$ bombardment does not induce a clear damage in the sample at least from a topographic perspective.

### 3.2. Core Level Analysis of the MWCNTs

An excellent technique to determine the density and the nature of defects present or produced in graphene-based materials, is core-level photoelectron spectroscopy. In fact, XPS is a very sensitive technique to finely determine the chemical bonding through the measured chemical shift in the C 1s core level components [34], like the sp${}^{2}$ and sp${}^{3}$ bonds, possible dangling bond states, etc.

**Figure 2:**
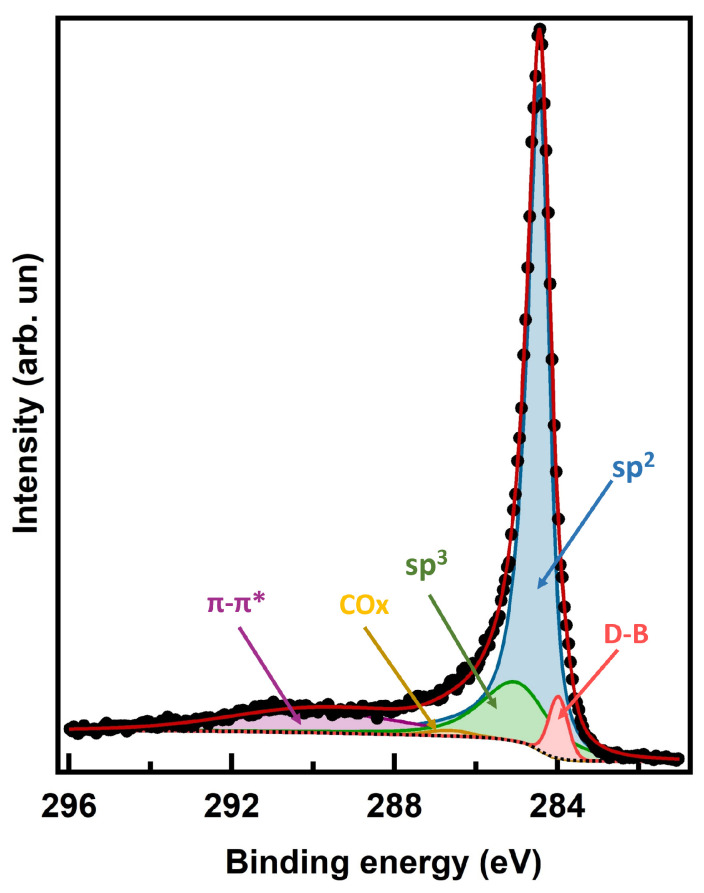
XPS C 1s core level of pristine CNT after annealing; experimental data (black dots), sp${}^{2}$ fitting component (blue area), sp${}^{3}$ component (green area), DB component (pink area), CO${}_{x}$ component (yellow area), π-plasmon component (violet area), Shirley background (dotted line) and fitting sum curve (red line).

The C 1s XPS spectrum of pristine MWCNTs taken after annealing to 400 °C is shown in Figure 2, along with the results of a fitting analysis. The experimental data have been deconvoluted using pseudo-Voigt line profiles (Lorentzian–Gaussian curves, with the Gaussian component taking into account the overall experimental uncertainty and the Lorentzian one the intrinsic excitation lifetime). We can single out five components: (i) the most intense associated to C−C sp${}^{2}$ bonds [34] at 284.5 eV BE; (ii) the one due to C−C sp${}^{3}$ distorted bonds at 285.1 eV (i.e., 0.6 eV higher BE than the sp${}^{2}$ peak), where the presence of sp${}^{3}$ bonds in pristine MWCNTs can be explained by the bending of the graphene surfaces in the nanotubes; (iii) an almost negligible component due to C-O${}_{x}$ bonds at 287.0 eV, related to residual oxygen contamination [19,35]; (iv) the expected π-plasmon (extended shake-up satellite) at 290.0 eV (i.e., 5.5 eV higher BE than the main peak); (v) a small fraction of dangling bonds (DB) component at low binding energy (283.9 eV) [36] that represents the tiny presence of vacancies in the MWCNTs.

The dominating sp${}^{2}$ component is estimated to be 66% w.r.t the overall peak intensity. The sp${}^{3}$ relative intensity ratio with respect to the sp${}^{2}$ is ∼19%, due to the bent nature of the cylindrical nanotubes [19,37,38,39,40]. We found very small concentration of oxygen, only 0.7% of overall intensity of the C 1s peak, in agreement with the measured intensity from the O 1s core level peak (see SI, Figure S4). The peak corresponding to vacancies in the honeycomb lattice, and associated to dangling bonds (DB), has a concentration of 3%. All fitting data of pristine MWCNTs are reported in the SI, Table S1.

**Figure 3:**
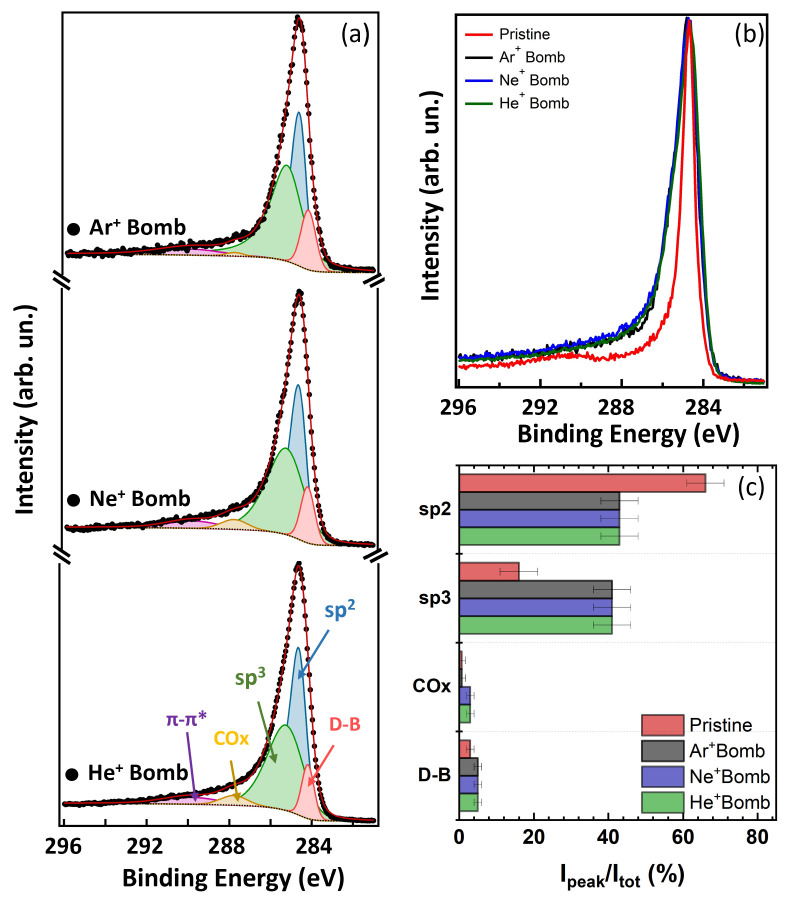
(**a**) XPS C 1s core level of the CNTs after ion bombardment with Ar${}^{+}$, Ne${}^{+}$ and He${}^{+}$ (spectra stacked from top to bottom, respectively); experimental data (black dots), sp${}^{2}$ fitting component (blue area), sp${}^{3}$ component (green area), DB component (pink area), CO${}_{x}$ component (yellow area), Shirley background (dotted line) and fitting sum curve (red line). (**b**) Experimental C 1s data for the pristine and bombarded CNTs. (**c**) Histogram of the percentage of the C 1s core level components for pristine clean MWCNTs (red bars), Ar${}^{+}$ bombarded CNTs (black bars), Ne${}^{+}$ bombarded CNTs (blue bars) and He${}^{+}$ bombarded CNTs (green bars); from the top to the bottom: sp${}^{2}$, sp${}^{3}$, CO${}_{x}$ and DB components.

Variation in the C 1s spectral lineshape of the bombarded MWCNTs with respect to the pristine sample is observed as an evident broadening, as shown in Figure 3b. The results of the fitting analysis are reported in the histogram of Figure 3c and in the SI Tables S2–S4.

After bombardment, both TOP and LAT, all the components do not shift in energy, while the sp${}^{2}$ peak and the π plasmon [20] are reduced in intensity, and the sp${}^{3}$ component representing the bond deformations in the lattice, shows a huge increase in intensity. The latter increase leads to a Θ ratio of about 48 ± 5%, where Θ = I(sp${}^{3}$)/[I(sp${}^{2}$) + I(sp${}^{3}$)]. Increase in dangling bond (from 3% to 8%) is also observed, due to an increase in vacancies in the honeycomb structure caused by bombardment. We do not observe any increase in the residual oxygen contamination onto the sample, thanks to the highly UHV controlled in situ experiment. XPS shows to be a very effective fingerprint of defect production in highly aligned MWCNTs after ion bombardment.

Finally, regarding the effect of different ion masses, we do not measure any significant difference in the C 1s spectral lineshapes, despite the different damage morphology observed by SEM. This can be explained by the local nature of defect production in the C mesh and on the very surface sensitivity of the XPS technique.

**Figure 4:**
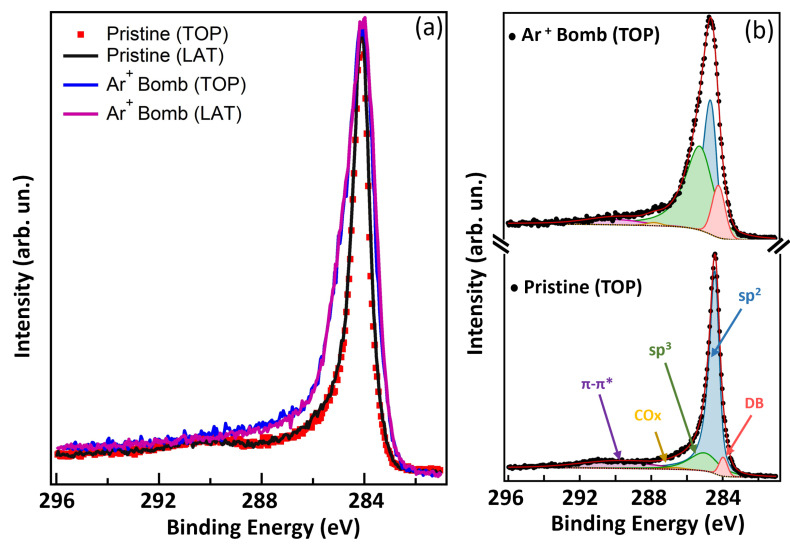
Comparative analysis of the XPS spectra of the CNTs after 3 keV Ar${}^{+}$ bombardment on top (TOP) and perpendicular to the CNT axes (LAT). (**a**) superimposed C 1s XPS experimental data for the clean pristine (red dots and black line) and Ar${}^{+}$ bombarded (blue and purple lines) CNTs. (**b**) fitting analysis of pristine TOP and Ar${}^{+}$ bombarded CNTs TOP; experimental data (black dots) with fitting curve components, namely sp${}^{2}$ (blue area), sp${}^{3}$ (green area), DB (pink area), CO${}_{x}$ (yellow area), Shirley background (dotted line) and fitting sum curve (red line).

We know from the SEM images that the morphology of CNTs is different from the TOP and LAT views, presenting circular voids from the top and elongated void parts among nanotubes on the side view. In order to obtain information about a possible spatial anisotropy in the defect production, we studied the XPS C 1s core level after bombardment of the two different sides of CNTs, TOP and LAT, as shown in Figure 4. To do these measurements we mounted two parts of the same sample oriented in the two sides (as shown in SI Figure S5), on the same sample holder, so to be able to bombard both sides at the same time in situ, with the same energy and dose.

We observe that there is not any lineshape change of the C 1s spectra of pristine CNTs taken for both orientations, as shown in Figure 4a. After bombarding with argon ions, we confirm the broadening of the lineshape, as discussed previously, but we do not observe any anisotropic effect depending on the nanotube orientation with respect to the impinging ion direction. This result confirms the local nature of the chemical damage induced by the ion beams, that does not depend on the spatial meso-scale morphology.

### 3.3. Raman Evidence of Defects

**Figure 5:**
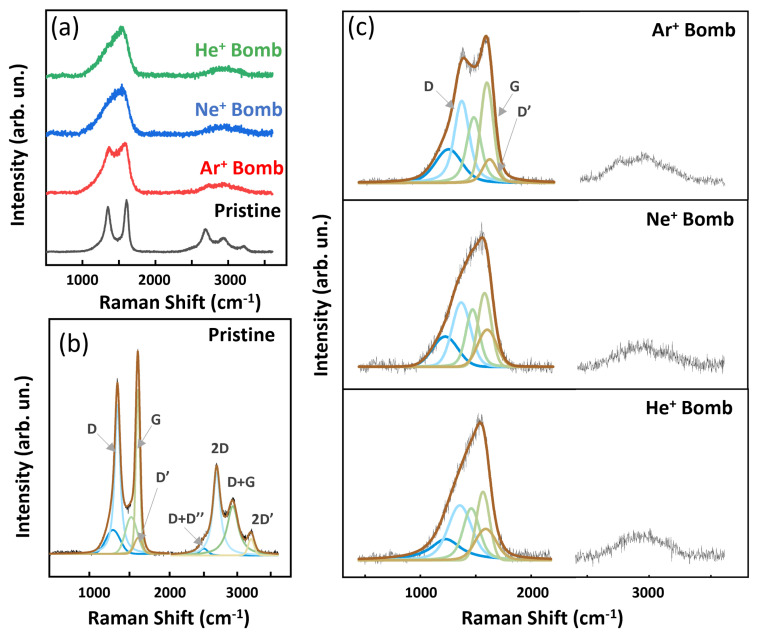
(**a**) Experimental data for Raman spectra (focused on the sample top) of Pristine and bombarded CNTs, (**b**) Fitted Raman spectra of pristine CNTs and Raman fitting components are reported with brown curves superimposed over the experimental data and (**c**) Fitted Raman spectra of the CNTs after ion bombardment with Ar${}^{+}$, Ne${}^{+}$ and He${}^{+}$ (spectra stacked from top to bottom, respectively).

Raman spectroscopy is one of the most efficient and well-known techniques to determine defects and lattice deformations in graphene-based materials [41]. The Raman spectrum of pristine MWCNTs taken on the top of the sample after annealing to 400 °C, shows five most prominent peaks, the D, G, 2D, D+G and 2D${}^{\prime }$ bands, all expected for MWCNTs [20,42], as shown in Figure 5b. We fitted the Raman bands with Lorentzian curves (fitted values are reported in SI Table S9). The G band is representative of C−C stretching mode and appears at 1604 cm${}^{-1}$, the D and D${}^{\prime }$ bands are associated with defects and distortions in the carbon hexagon rings and they appear at 1350 cm${}^{-1}$ and 1610 cm${}^{-1}$, respectively, and the 2D band appears at 2690 cm${}^{-1}$. Bumpy broad structures due to some residual amorphous carbon signal are considered below the main peaks by using Gaussian curves, in agreement with the literature on MWCNTs [19]. The integrated intensity ratios between the D and G band I(D)/I(G), and between the 2D and G bands I(2D)/I(G) are 0.90 and 0.47, respectively. The peak widths of the different peaks are 60 cm${}^{-1}$, 81 cm${}^{-1}$, 116 cm ${}^{-1}$, 145 cm${}^{-1}$, 132 cm${}^{-1}$, 198 cm${}^{-1}$ and 109 cm${}^{-1}$ for the G, D, D${}^{\prime }$, D+D${}^{\prime \prime }$, 2D, D+G and 2D${}^{\prime }$ bands, respectively.

Raman data taken on the top of the He${}^{+}$, Ne${}^{+}$ and Ar${}^{+}$ bombarded CNTs, as compared to the pristine one, are shown in Figure 5a. We fitted the Raman bands and fitting results are reported in Figure 5c. We observe a clear broadening of all main Raman bands, and also an important increase of the Gaussian component underlying the peaks, associated to the amorphous response [43,44]. In particular, broadening of the G and D bands after bombardment reaches 140 cm${}^{-1}$ and 253 cm${}^{-1}$, respectively, (see the SI Tables S10–S12), due to the induction of defects by noble gas ions hitting the MWCNTS. A frequency shift is observed up to 1357 cm${}^{-1}$ for the D band and decrease down to 1565 cm${}^{-1}$ for the G band, probably associated to strain induced by defects introduced after bombardment. In the mid-range between these two peaks, there is a broad band at 1460 cm${}^{-1}$ related to increase in amorphous carbon [45]. Looking at the ion mass dependence of the produced lattice damage, it is evident that the lighter mass ions cause more diffused lattice damage than the heavier ones, as observable through the relative increasing broadening (values in the SI, Tables S10–S12) upon going from Ar${}^{+}$, to Ne${}^{+}$ eventually to He${}^{+}$ ions. The results of Raman study correlate well with the SEM data.

The Raman and SEM results suggest the following picture of produced damage: the energy loss of ions to CNT atomic electrons at velocity less than 0.01 c (where c is the speed of light in vacuum) can be described by Lindhard’s model [46,47]. This model predicts an energy loss increasing with the ion velocity, as the data of CNT damage suggest: in fact, He ions are the fastest (∼3.8 × 10${}^{5}$ m/s), they easily reach a noticeable depth and consequently produce a more diffuse damage of the CNT lattice, while the Ar ions produce more localized defects in the lattice.

## 4. Conclusions

The effects of different masses of noble gas in ion irradiation on the chemical state, lattice morphology and local structure of MWCNTs were studied via XPS, Raman and SEM. It has been established that bombardment with ions on different geometries (TOP and LAT) of MWCNTs leads to defects and distortions in the MWCNT’s structure. In particular, bundles and clustering of CNTs are clearly visible in the SEM images, where smaller voids on the top are produced by lighter ions. Raman analysis confirms the SEM results, showing lattice damage in the CNTs, as observed by broadening of Raman bands accompanied by partial amorphization.

The Raman spectroscopy data show more diffused lattice defects by bombarding with lighter ions (He${}^{+}$) than heavier ones (Ar${}^{+}$). The interaction of the heavier ions with the C lattice produces more localized defects; as a consequence, the damage depth reached by Ar is smaller than for He, the latter producing diffuse lattice defects.

The analysis of the C 1s core level spectra has shown an increase in the fraction of C atoms with sp${}^{3}$ hybridized bonds from 19 ± 5% up to 48 ± 5%, and a reduction of the π-plasmon of delocalized electrons on the surface of MWCNTs. Furthermore, not any anisotropic effect is observed in the XPS study (TOP or LAT bombardment), due to very local, atomic and surface sensitive characteristics of XPS.

These ion-bombarded MWCNTs, where we demonstrated how to increase available sp${}^{3}$ bonds thanks to clean and controlled UHV in situ ion bombardment, can constitute excellent scaffolds for further functionalization. For example, sp${}^{3}$ bonds may strongly favor the uploading of hydrogen, offering highly useful and potential applications in new solid-state materials for energy storage.

## Data Availability

Data are contained within the article.

## Supplementary Materials

The following are available online at https://www.mdpi.com/article/10.3390/nano14010077/s1, Figure S1: Exemplary SEM images of MWCNTs from (a) (LAT) view and (b) (TOP) view, used for size estimation by the Gwyddion program.; Figure S2: mean hole size at the top of the MWCNT after ion bombardment; Figure S3: analysis of the SEM images to determine the mean hole size; Figure S4: survey XPS spectrum at pristine MWCNTs; Figure S5: MWCNTs mounted from TOP and LAT sides on same sample holder; Figure S6: FWHM evolution of Raman bands (D, G and D${}^{\prime }$) for Pristine and Bombarded MWCNTs (Ar${}^{+},$ Ne${}^{+}$ and He${}^{+}$) after fitting analysis.; Table S1: C1s fitting parameters from the C1s core level of the pristine MWCNT sample; Tables S2–S4: C1s fitting parameters from the C1s core levels of the Ar-, Ne-, and He-bombarded MWCNT samples; Tables S5 and S6: C1s fitting parameters from the C1s core levels of the TOP and LAT pristine sample; Tables S7 and S8: C1s fitting parameters from the C1s core levels of the TOP and LAT Ar-bombarded sample; Table S9: Raman band fitting parameters for pristine MWCNT; Tables S10–S12: Raman band fitting parameters for Ar, Ne, He bombareded MWCNT. Reference [48] is cited in the Supplementary Materials.

## Abbreviations

The following abbreviations are used in this manuscript:

CNT	Carbon nanotube
MWCNT	Multi Wall Carbon nanotube
VA-MWCNT	Vertically Aligned Multi Wall Carbon nanotube
XPS	X ray Photoelectron Spectroscopy
UHV	Ultra High Vacuum
SEM	Scanning Electron microscopy
3D	Three Dimensional
2D	Two Dimensional
1D	One Dimensional

## References

[1] Iijima S. (1991). Helical microtubules of graphitic carbon. Nature.

[2] Andrews R., Jacques D., Rao A., Derbyshire F., Qian D., Fan X., Dickey E., Chen J. (1999). Continuous production of aligned carbon nanotubes: A step closer to commercial realization. Chem. Phys. Lett..

[3] Ma Y., Foster A.S., Krasheninnikov A., Nieminen R.M. (2005). Nitrogen in graphite and carbon nanotubes: Magnetism and mobility. Phys. Rev. B.

[4] Nakaharai S., Iwasaki T., Morita Y., Moriyama S., Ogawa S. (2022). Electron transport tuning of graphene by helium ion irradiation. Nano Express.

[5] Ogawa S. (2016). Anderson localization of graphene by helium ion irradiation. Appl. Phys. Lett..

[6] Pejcic B., Myers M., Ranwala N., Boyd L., Baker M., Ross A. (2011). Modifying the response of a polymer-based quartz crystal microbalance hydrocarbon sensor with functionalized carbon nanotubes. Talanta.

[7] Zhao Q., Yuan Z., Duan Z., Jiang Y., Li X., Li Z., Tai H. (2019). An ingenious strategy for improving humidity sensing properties of multi-walled carbon nanotubes via poly-L-lysine modification. Sens. Actuators B Chem..

[8] Star A., Gabriel J.C.P., Bradley K., Grüner G. (2003). Electronic detection of specific protein binding using nanotube FET devices. Nano Lett..

[9] Wong M., Paramsothy M., Xu X., Ren Y., Li S., Liao K. (2003). Physical interactions at carbon nanotube-polymer interface. Polymer.

[10] Rakhi R., Sethupathi K., Ramaprabhu S. (2008). Field emission from carbon nanotubes on a graphitized carbon fabric. Carbon.

[11] Lee Y.H., An K.H., Lim S.C., Kim W.S., Jeong H.J., Doh C.H., Moon S.I. (2002). Applications of carbon nanotubes to energy storage devices. New Diam. Front. Carbon Technol..

[12] Rivard E., Trudeau M., Zaghib K. (2019). Hydrogen storage for mobility: A review. Materials.

[13] Wang B., Pang B. (2020). Properties improvement of multiwall carbon nanotubes-reinforced cement-based composites. J. Compos. Mater..

[14] Tans S.J., Verschueren A.R.M., Dekker C. (1998). Room-temperature transistor based on a single carbon nanotube. Nature.

[15] Derakhshandeh J., Abdi Y., Mohajerzadeh S., Hosseinzadegan H., Soleimani E.A., Radamson H. (2005). Fabrication of 100 nm gate length MOSFET’s using a novel carbon nanotube-based nano-lithography. Mater. Sci. Eng. B.

[16] Raffaelle R., Landi B., Harris J., Bailey S., Hepp A. (2005). Carbon nanotubes for power applications. Mater. Sci. Eng. B.

[17] An K.H., Kim W.S., Park Y.S., Choi Y.C., Lee S.M., Chung D.C., Bae D.J., Lim S.C., Lee Y.H. (2001). Supercapacitors using single-walled carbon nanotube electrodes. Adv. Mater..

[18] Scardamaglia M., Amati M., Llorente B., Mudimela P., Colomer J.F., Ghijsen J., Ewels C., Snyders R., Gregoratti L., Bittencourt C. (2014). Nitrogen ion casting on vertically aligned carbon nanotubes: Tip and sidewall chemical modification. Carbon.

[19] D’Acunto G., Ripanti F., Postorino P., Betti M.G., Scardamaglia M., Bittencourt C., Mariani C. (2018). Channelling and induced defects at ion-bombarded aligned multiwall carbon nanotubes. Carbon.

[20] Lehtinen O., Nikitin T., Krasheninnikov A.V., Sun L., Banhart F., Khriachtchev L., Keinonen J. (2011). Characterization of ion-irradiation-induced defects in multi-walled carbon nanotubes. New J. Phys..

[21] Mathew S., Bhatta U., Joseph B., Dev B. (2007). keV Ag ion irradiation induced damage on multiwalled carbon nanotubes. Nucl. Instrum. Methods Phys. Res. Sect. B Beam Interact. Mater. Atoms.

[22] Krasheninnikov A., Nordlund K. (2005). Channeling of heavy ions through multi-walled carbon nanotubes. Nucl. Instrum. Methods Phys. Res. Sect. B Beam Interact. Mater. Atoms.

[23] Salonen E., Krasheninnikov A., Nordlund K. (2002). Ion-irradiation-induced defects in bundles of carbon nanotubes. Nucl. Instrum. Methods Phys. Res. Sect. B Beam Interact. Mater. Atoms.

[24] Krasheninnikov A., Nordlund K. (2004). Irradiation effects in carbon nanotubes. Nucl. Instrum. Methods Phys. Res. Sect. B Beam Interact. Mater. Atoms.

[25] Buchowicz G., Stone P.R., Robinson J.T., Cress C.D., Beeman J.W., Dubon O.D. (2011). Correlation between structure and electrical transport in ion-irradiated graphene grown on Cu foils. Appl. Phys. Lett..

[26] Betti M.G., Placidi E., Izzo C., Blundo E., Polimeni A., Sbroscia M., Avila J., Dudin P., Hu K., Ito Y. (2022). Gap Opening in Double-Sided Highly Hydrogenated Free-Standing Graphene. Nano Lett..

[27] Junge F., Auge M., Zarkua Z., Hofsäss H. (2023). Lateral Controlled Doping and Defect Engineering of Graphene by Ultra-Low-Energy Ion Implantation. Nanomaterials.

[28] Marchiani D., Tonelli A., Mariani C., Frisenda R., Avila J., Dudin P., Jeong S., Ito Y., Magnani F.S., Biagi R. (2023). Tuning the Electronic Response of Metallic Graphene by Potassium Doping. Nano Lett..

[29] Schifano E., Cavoto G., Pandolfi F., Pettinari G., Apponi A., Ruocco A., Uccelletti D., Rago I. (2023). Plasma-Etched Vertically Aligned CNTs with Enhanced Antibacterial Power. Nanomaterials.

[30] Sarasini F., Tirillò J., Lilli M., Bracciale M.P., Vullum P.E., Berto F., De Bellis G., Tamburrano A., Cavoto G., Pandolfi F. (2022). Highly aligned growth of carbon nanotube forests with in situ catalyst generation: A route to multifunctional basalt fibres. Compos. Part B Eng..

[31] (2023). SmartLab. https://sites.google.com/uniroma1.it/smartlab.

[32] Abdelnabi M.M.S., Izzo C., Blundo E., Betti M.G., Sbroscia M., Di Bella G., Cavoto G., Polimeni A., García-Cortés I., Rucandio I. (2021). Deuterium Adsorption on Free-Standing Graphene. Nanomaterials.

[33] Vincent L., Soille P. (1991). Watersheds in digital spaces: An efficient algorithm based on immersion simulations. IEEE Trans. Pattern Anal. Mach. Intell..

[34] Lacovig P., Pozzo M., Alfe D., Vilmercati P., Baraldi A., Lizzit S. (2009). Growth of dome-shaped carbon nanoislands on Ir (111): The intermediate between carbidic clusters and quasi-free-standing graphene. Phys. Rev. Lett..

[35] Estrade-Szwarckopf H. (2004). XPS photoemission in carbonaceous materials: A “defect” peak beside the graphitic asymmetric peak. Carbon.

[36] Susi T., Kaukonen M., Havu P., Ljungberg M.P., Ayala P., Kauppinen E.I. (2014). Core level binding energies of functionalized and defective graphene. Beilstein J. Nanotechnol..

[37] Díaz J., Paolicelli G., Ferrer S., Comin F. (1996). Separation of the *sp*^3^ and *sp*^2^ components in the C1*s* photoemission spectra of amorphous carbon films. Phys. Rev. B.

[38] Chu P.K., Li L. (2006). Characterization of amorphous and nanocrystalline carbon films. Mater. Chem. Phys..

[39] Sun C.Q., Sun Y., Nie Y.G., Wang Y., Pan J.S., Ouyang G. (2009). Coordination-Resolved C-C Bond Length and the C 1s Binding Energy of Carbon Allotropes and the Effective Atomic Coordination of the Few-Layer Graphene. J. Phys. Chem. C.

[40] Al-Harthi S.H., Elzain M., Al-Barwani M., Kora’a A., Hysen T., Myint M.T.Z., Anantharaman M.R. (2012). Unusual surface and edge morphologies, sp2 to sp3 hybridized transformation and electronic damage after Ar+ ion irradiation of few-layer graphene surfaces. Nanoscale Res. Lett..

[41] Malard L., Pimenta M., Dresselhaus G., Dresselhaus M. (2009). Raman spectroscopy in graphene. Phys. Rep..

[42] Antunes E., Lobo A., Corat E., Trava-Airoldi V. (2007). Influence of diameter in the Raman spectra of aligned multi-walled carbon nanotubes. Carbon.

[43] Antunes E., Lobo A., Corat E., Trava-Airoldi V., Martin A., Veríssimo C. (2006). Comparative study of first- and second-order Raman spectra of MWCNT at visible and infrared laser excitation. Carbon.

[44] Jeet K., Jindal V.K., Bharadwaj L.M., Avasthi D.K., Dharamvir K. (2010). Damaged carbon nanotubes get healed by ion irradiation. J. Appl. Phys..

[45] Ferrari A.C., Robertson J. (2000). Interpretation of Raman spectra of disordered and amorphous carbon. Phys. Rev. B.

[46] Lindhard J. (1954). On the properties of a gas of charged particles. Kgl. Danske Videnskab. Selskab Mat. Fys. Medd..

[47] Lindhard J., Scharff M., Schioett H.E. (1963). Range Concepts and Heavy Ion Ranges (Notes on Atomic Collisions, II). Kgl. Danske Videnskab. Selskab. Mat. Fys. Medd..

[48] Yeh J., Lindau I. (1985). Atomic subshell photoionization cross sections and asymmetry parameters: 1 ≤ *Z* ≤ 103. At. Data Nucl. Data Tables.

