# Supplementary material for: Spectromicroscopy Study of Induced Defects in Ion-Bombarded Highly Aligned Carbon Nanotubes
Source: Nanomaterials (Basel). 2023 Dec 27;14(1):77. doi: 10.3390/nano14010077 (PMC10780812; doi:10.3390/nano14010077)
Supplement: Supplementary file 1 [file nanomaterials-14-00077-s001.zip › nanomaterials-2786416-supplementary.pdf]

Article

Supplementary Information: Spectromicroscopy study of induced defects in ion-bombarded highly aligned carbon nanotubes

Sammar Tayyab[1,2]*, Alice Apponi[3], Maria Grazia Betti[1,2], Elena Blundo[1], Gianluca Cavoto[1,2], Riccardo Frisenda[1], Nuria Jiménez-Arévalo[1], Carlo Mariani[1,2]*, Francesco Pandolfi[2], Antonio Polimeni[1], Ilaria Rago[1,2], Alessandro Ruocco[3], Marco Sbroscia[1], Ravi Prakash Yadav[1,2]

1 Dipartimento di Fisica, Sapienza Università di Roma, P.le Aldo Moro 2, 00185 Rome, Italy
2 INFN Rome, Piazzale Aldo Moro 2, 00185 Rome, Italy
3 Dipartimento di Scienze, Università Degli Studi Roma Tre and INFN Sezione di Roma Tre, Via della Vasca Navale 84, 00146 Rome, Italy
* Corresponding authors: sammar.tayyab@uniroma1.it; Tel.: +39-06-49914281 (S.T.) and carlo.mariani@uniroma1.it; Tel.: +39-06-49914393 (C.M.)

Version December 26, 2023 submitted to Nanomaterials

SEM Analysis

From the set of SEM images of the used samples, we estimated the average heights and diameters by using the Gwyddion program. We obtained heights of 190 $\pm$ 5 μm, 209 $\pm$ 5 μm and 181 $\pm$ 5 μm at the pristine CNTs used for Ar, Ne and He bombardment, respectively. The corresponding average diameters are 11 nm, 10 nm and 13 nm.

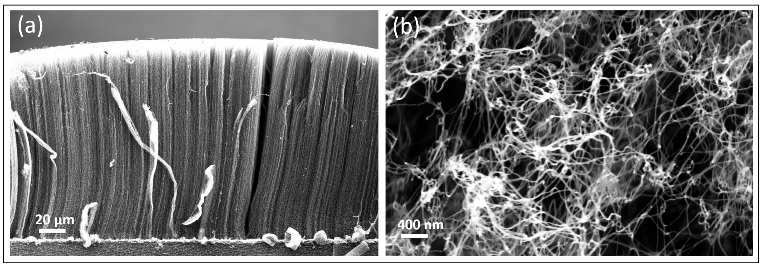

Figure S1. Exemplary SEM images of MWCNTs from (a)(LAT) view and (b)(TOP) view, used for size estimation by the Gwyddion program.

The SEM images taken on the pristine and noble gas ion bombarded samples (plotted in Figure 1 of the main text), have been analyzed in order to get an average value and a distribution of the produced hole size. The analysis of the holes caused by bombardment in the TOP samples has been done applying a grain size analysis based on a watershed algorithm [1] and using the image processing software Gwyddion. In Figure S2, we show three examples of analyzed images with the detected holes presented as a masks in red color.

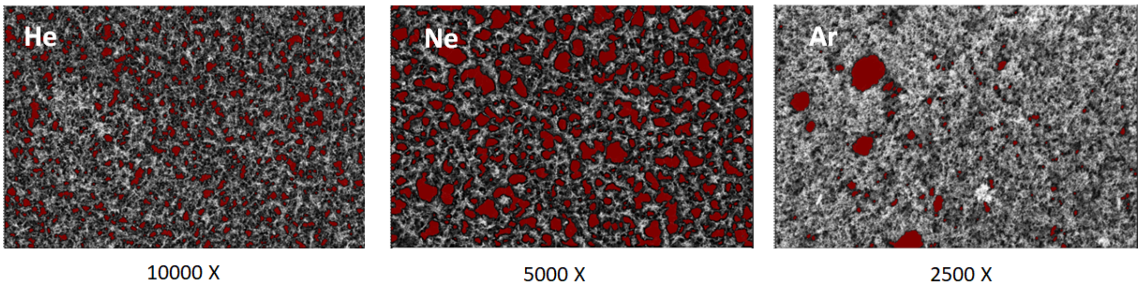

Figure S2. Hole size detection analysis from the SEM images elaboration of the He^+, Ne^+ and Ar^+ bombarded MWCNTs; each red spot is a different found grain corresponding to holes in the SEM image.

The analysis gives an almost exponential distribution of voids, we estimate it as the 90th percentile, i.e. largest 10% of the area distribution. This estimates top 10% grain area for the four samples is shown in Figure S3.

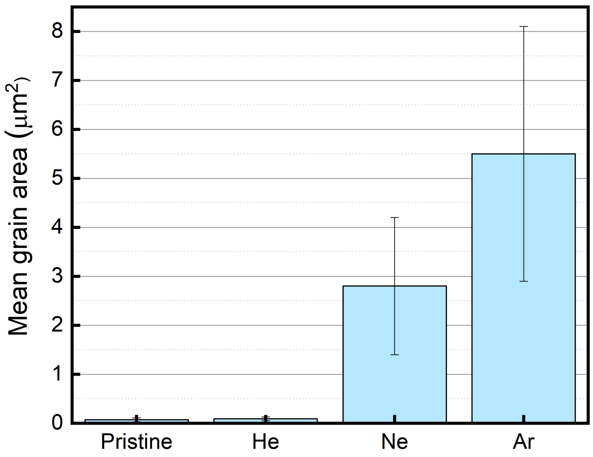

Figure S3. Mean hole size of the TOP of pristine and ion-bombarded CNTs, as estimated from the SEM images.

Survey XPS data of the pristine MWCNT sample

An XPS survey spectrum of the pristine MWCNT in the range of 0 - 600 eV binding energy is plotted in Figure S4. It can be seen that the sample is composed of Carbon (C), as expected, and a negligible amount of oxygen (O). We estimated the amount of oxygen present in the sample by taking into account the excitation cross-sections of C1s and O1s core levels [2], obtaining only 0.7% of O content.

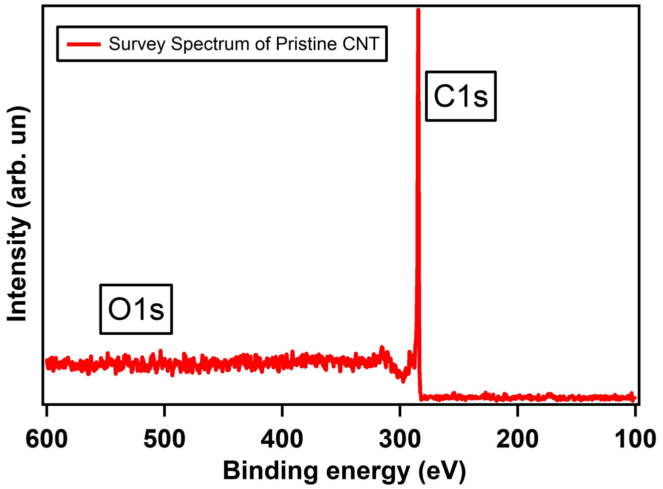

Figure S4. XPS survey spectrum of pristine CNT after annealing.

XPS fitting procedure

The XPS C1s data for the pristine MWCNT and for the ion bombarded MWCNT samples have been fit by pseudo-Voigt line profiles (Lorentzian-Gaussian curves, with the Gaussian component taking into account the overall experimental uncertainty and the Lorentzian one the intrinsic excitation lifetime). The Lorenztian component presents an asymmetry for the sp^2 peak [3], due to the metallic tail of CNTs, associated to the continuous transitions across the Fermi level in graphene-based materials. The fitting results plotted in the main paper for the pristine MWCNT sample are reported in Table S1.

Table S1. Fit results for the C1s core level of pristine MWCNT sample. Binding Energy (B.E, ± 0.1 eV), full width at half-maximum (FWHM, ± 0.1eV), $\Theta = I(sp^3)/[I(sp^2) + I(sp^3)]$.

C1s component	BE (eV)	Δ BE (eV)	FWHM (eV)	$Area/Area_{total}$	Θ (%)
sp^2	284.5	0	0.7	66	19
sp^3	285.1	0.6	2.0	16	
COx	287.0	2.5	1.2	0.7	
π - excitation	290.0	5.5	5.0	11	
vacancy - DB	283.9	-0.6	0.5	3	

The sp^2 component presents a well defined bonding state of Carbon atoms, width of the peak is related to experimental resolution. The sp^2 component is fitted with asymmetric Lorentzian component with 0.2 asymmetry parameter; this function is considered due to metallic nature of sp^2 peak in graphene- like lattice, with the formation of core-hole pairs [3]. The lineshape of this component as shown in Figure S2 represents a good quality of carbon honeycomb lattice in MWCNT structure. The sp^3 component is due to the distorted carbon bonds in the lattice. The plasmon represents the multiple excitations of CNTs and this component is 5.5 eV away from the main peak.

XPS analysis of ion bombardmed samples

The C1s fittings values after Ar^+, Ne^+ and He^+ ion bomardment on MWCMNTs at 3 keV energy are reported in the Tables S2, S3 and S4, respectively.

Table S2. Fit results for the C1s core level of Ar^+ bombarded MWCNT sample. Binding Energy (B.E, ± 0.1 eV), full width at half-maximum (FWHM, ± 0.1eV), $\Theta = I(sp^3)/[I(sp^2) + I(sp^3)]$.

C1s component	BE (eV)	Δ BE (eV)	FWHM (eV)	$Area/Area_{total}$	Θ (%)
sp^2	284.7	0	1.0	43	48
sp^3	285.3	0.6	2.1	41	
COx	287.7	3.0	1.2	0.7	
π - excitation	290.0	5.3	3.1	5	
vacancy - DB	284.2	-0.5	0.7	8	

Table S3. Fit results for the C1s core level of Ne^+ bombarded MWCNT sample. Binding Energy (B.E, ± 0.1 eV), full width at half-maximum (FWHM, ± 0.1 eV), $\Theta = I(sp^3)/[I(sp^2) + I(sp^3)]$.

C1s component	BE (eV)	Δ BE (eV)	FWHM (eV)	$Area/Area_{total}$	Θ (%)
sp^2	284.7	0	1.0	43	48
sp^3	285.3	0.6	2.1	41	
COx	287.7	3.0	1.5	3	
π - excitation	290.0	5.3	3.1	5	
vacancy - DB	284.2	-0.5	0.7	8	

Table S4. Fit results for the C1s core level of He^+ bombarded MWCNT sample. Binding Energy (B.E, ± 0.1 eV), full width at half-maximum (FWHM, ± 0.1eV), $\Theta = I(sp^3)/[I(sp^2) + I(sp^3)]$.

C1s component	BE (eV)	Δ BE (eV)	FWHM (eV)	$Area/Area_{total}$	Θ (%)
sp^2	284.7	0	1.0	43	48
sp^3	285.3	0.6	2.1	41	
COx	287.7	3.0	1.5	3	
π - excitation	290.0	5.3	3.1	5	
vacancy - DB	284.2	-0.5	0.7	8	

XPS analysis of Pristine and ion bombarded TOP and LAT samples

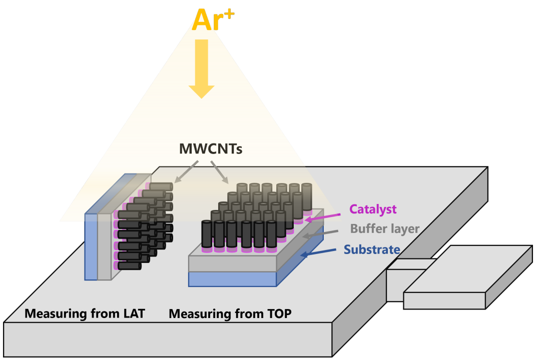

Figure S5. MWCNTs mounted from TOP and LAT sides on same sample holder for micro-XPS measurements.

Table S5. Fit results for the C1s core level of TOP side of pristine MWCNT sample. Binding Energy (B.E, ± 0.1 eV), full width at half-maximum (FWHM, ± 0.1eV), Θ = I(sp^3)/[I(sp^2) + I(sp^3)].

C1s component	BE (eV)	Δ BE (eV)	FWHM (eV)	Area/$Area_{total}$	Θ (%)
sp^2	284.5	0	0.7	66	19
sp^3	285.1	0.6	2.0	16	
COx	287.0	2.5	1.2	0.7	
π - excitation	290.0	5.5	5.0	11	
vacancy - DB	283.9	-0.6	0.5	3	

Table S6. Fit results for the C1s core level of LAT side of pristine MWCNT sample. Binding Energy (B.E, ± 0.1 eV), full width at half-maximum (FWHM, ± 0.1eV), Θ = I(sp^3)/[I(sp^2) + I(sp^3)].

C1s component	BE (eV)	Δ BE (eV)	FWHM (eV)	Area/$Area_{total}$	Θ (%)
sp^2	284.5	0	0.7	66	19
sp^3	285.1	0.6	2.0	16	
COx	287.0	2.5	1.2	0.7	
π - excitation	290.0	5.5	5.0	11	
vacancy - DB	283.9	-0.6	0.5	3	

Table S7. Fit results for the C1s core level of TOP Ar^+ bombarded MWCNT sample. Binding Energy (B.E, ± 0.1 eV), full width at half-maximum (FWHM, ± 0.1eV), Θ = I(sp^3)/[I(sp^2) + I(sp^3)].

C1s component	BE (eV)	Δ BE (eV)	FWHM (eV)	Area/$Area_{total}$	Θ (%)
sp^2	284.7	0	1.0	43	48
sp^3	285.3	0.6	2.1	41	
COx	287.7	3.0	1.2	0.7	
π - excitation	290.0	5.3	3.1	5	
vacancy - DB	284.2	-0.5	0.7	8	

Table S8. Fit results for the C1s core level of LAT Ar^+ bombarded MWCNT sample. Binding Energy (B.E, ± 0.1 eV), full width at half-maximum (FWHM, ± 0.1eV), Θ = I(sp^3)/[I(sp^2) + I(sp^3)].

C1s component	BE (eV)	Δ BE (eV)	FWHM (eV)	Area/$Area_{total}$	Θ (%)
sp^2	284.7	0	1.0	43	48
sp^3	285.3	0.6	2.1	41	
COx	287.7	3.0	1.2	0.7	
π - excitation	290.0	5.3	3.1	5	
vacancy - DB	284.2	-0.5	0.7	8	

Raman Data and fitting analysis of the pristine and ion bombarded MWCNTs.

The Raman spectrum of pristine MWCNTs after annealing to 400°C shows five most prominent peaks, the D, G, 2D, D+G and 2D′ bands expected for MWCNTs, as shown in the figure of main paper. We fitted the main Raman bands with Lorentzian curves and all wide bumps were fitted with Gaussian curves. The G band is representative of C-C stretching mode and appears at 1604 cm^{-1}, D and D′ bands are purely associated with defects and distortions in the carbon hexagon rings and they appear at 1350 cm^{-1} and 1610 cm^{-1}, respectively, the 2D band appears at 2690 cm^{-1}. Bumpy broad structures are considered below the main peaks by using Gaussian curves, in agreement with the literature on MWCNTs [4]. Fitting results on the pristine sample are reported in the Table S9.

Table S9. Fit results of the Raman data of the pristine MWCNT sample. All data but the areas with 5% precision.

Peak	Fitting curve	Wavenumber (cm^{-1})	FWHM (cm^{-1})	Intensity (Area)
bump	Gaussian	1294	207	24
D	Lorenztian	1350	81	85
bump	Gaussian	1521	172	31
G	Lorenztian	1604	60	53
D′	Lorenztian	1610	116	9
D+D″	Lorenztian	2498	145	6
2D	Lorenztian	2690	132	80
D+G	Lorenztian	2932	198	72
2D′	Lorenztian	3206	109	9

The Raman data after bombardment indicate deep modifications that can be represented by broad Raman band widths. The result of the fitting analysis after ion bombardment are reported in Table S10, S11 and S12.

Table S10. Fit results of the Raman data of the Ar^+ bombarded MWCNT sample. All data but the areas with 5% precision;[E_{Ar+} = 3000 eV]

Peak	Fitting curve	Wavenumber (cm^{-1})	FWHM (cm^{-1})	Intensity (Area)
bump	Gaussian	1240	281	72
D	Lorenztian	1357	159	112
bump	Gaussian	1465	152	82
G	Lorenztian	1580	134	98
D′	Lorenztian	1605	148	27

Table S11. Fit results of the Raman data of the Ne^+ bombarded MWCNT sample. All data but the areas with 5% precision; [E_{Ne+} = 3000 eV]

Peak	Fitting curve	Wavenumber (cm^{-1})	FWHM (cm^{-1})	Intensity (Area)
bump	Gaussian	1214	266	54
D	Lorenztian	1357	195	89
bump	Gaussian	1459	142	61
G	Lorenztian	1566	141	70
D′	Lorenztian	1590	198	50

Table S12. Fit results of the Raman data of the He^+ bombarded MWCNT sample. All data but the areas with 5% precision; [E_{He+} = 3000 eV]

Peak	Fitting curve	Wavenumber (cm^{-1})	FWHM (cm^{-1})	Intensity (Area)
bump	Gaussian	1230	332	70
D	Lorenztian	1357	253	110
bump	Gaussian	1460	176	74
G	Lorenztian	1565	140	65
D′	Lorenztian	1590	224	52

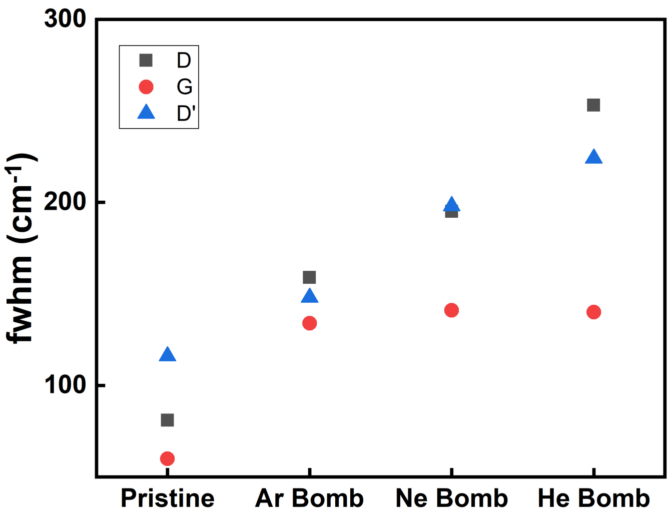

Figure S6. FWHM evolution of Raman bands (D, G and D') for Pristine and Bombarded MWCNTs (Ar^+,Ne^+ and He^+) after fitting analysis.

References

1. Vincent, L.; Soille, P. Watersheds in digital spaces: an efficient algorithm based on immersion simulations. *IEEE Transactions on Pattern Analysis and Machine Intelligence* **1991**, *13*, 583–598. doi:10.1109/34.87344.
2. Yeh, J.; Lindau, I. Atomic subshell photoionization cross sections and asymmetry parameters: $1 \leq Z \leq 103$. *Atomic data and nuclear data tables* **1985**, *32*, 1–155.
3. Lacovig, P.; Pozzo, M.; Alfe, D.; Vilmercati, P.; Baraldi, A.; Lizzit, S. Growth of dome-shaped carbon nanoislands on Ir (111): the intermediate between carbidic clusters and quasi-free-standing graphene. *Physical Review Letters* **2009**, *103*, 166101.
4. D'Acunto, G.; Ripanti, F.; Postorino, P.; Betti, M.G.; Scardamaglia, M.; Bittencourt, C.; Mariani, C. Channelling and induced defects at ion-bombarded aligned multiwall carbon nanotubes. *Carbon* **2018**, *139*, 768 – 775. doi:https://doi.org/10.1016/j.carbon.2018.07.032.
